# Evaluation of serum interleukin-17 A and interleukin-22 levels in pediatric patients with autism spectrum disorder: a pilot study

**DOI:** 10.1186/s12887-023-04484-2

**Published:** 2024-01-05

**Authors:** Dina E. Sallam, Youstina S. Shaker, Gehan A. Mostafa, Reham M. El-Hossiny, Sara I. Taha, Mostafa Abd Elazeem Hassan Ahamed

**Affiliations:** 1 Department of Pediatrics, Pediatric Nephrology Unit, Faculty of Medicine, Ain Shams University, Abbasia, Cairo, Egypt; 2 Faculty of Medicine, Ain Shams University, Cairo, Egypt; 3 Department of Pediatrics, Pediatric Allergy, and Immunology Unit, Faculty of Medicine, Ain Shams University, Cairo, Egypt; 4 Department of Pediatrics, Pediatric Neuropsychiatric Unit, Faculty of Medicine, Ain Shams University, Cairo, Egypt; 5 Department of Clinical Pathology, Faculty of Medicine, Ain Shams University, Cairo, Egypt; 6 Department of Pediatrics, Faculty of Medicine, Al Azhar University, Cairo, Egypt

**Keywords:** Autism, Cytokines, Interleukin, Neurodevelopment, Pediatric

## Abstract

**Background:**

Many neurodevelopmental abnormalities are connected to autism spectrum disorder (ASD), which can result in inflammation and elevated cytokine levels due to immune system dysregulation. Interleukin (IL)-17 A and IL-22 have been linked to the regulation of host defense against pathogens at the barrier surface, the regeneration of injured tissue, and the integration of the neurological, endocrine, and immune systems. Several studies have investigated the possible connection between IL-17 A and ASD as well as the severity of behavioral symptoms, but few of them included IL-22.

**Objectives:**

To measure serum levels of interleukin (IL)-17 A and IL-22 in children with ASD and to investigate their association with disease severity.

**Methods:**

This pilot study was performed on 24 children with ASD and 24 matched controls. Childhood Autism Rating Scale (CARS) assessed ASD severity, and serum levels of IL-17 A and IL-22 were assessed by enzyme-linked immunosorbent assay (ELISA).

**Results:**

In ASD patients, serum levels of IL-17 A and IL-22 showed a significant increase compared to controls (*p*-values < 0.001). We compared serum levels of IL-17 A and IL-22 according to the severity categories by CARS and could not find any significant differences (*p*-values > 0.05). Only IL-22 had a significant positive correlation with ASD severity by CARS scores.

**Conclusions:**

Raised serum levels of IL-17 A and IL-22 are associated with ASD; only IL-22, not IL-17 A, is correlated with ASD severity. This finding proposes IL-22 as a possible future effective target for ASD treatment. To fully comprehend the significance of these cytokines in ASD and their possible effects on ASD diagnosis and treatment, more research on a wider scale is required.

## Introduction

Autism spectrum disorder (ASD) is a set of neurodevelopmental abnormalities of varying symptoms and severity associated with difficulties in social interaction and communication [1]. It has no specific cause; genetics and environmental factors may play a role [2]. There is growing evidence that the immune system is essential in developing ASD. Some autistic children have brain-specific autoantibodies, which may indicate central nervous system (CNS) autoimmunity. Also, the increased incidence of autoimmune illness in some autistic families is another indication of the autoimmune pathogenesis of autism [3, 4]. Moreover, some autistic children may have increased lymphocytes with an imbalance in T-helper (Th) cell subsets, which could damage astrocytes and disrupt the blood-brain barrier (BBB) [5]. Th17 cells, a unique subset of effector ${\text{C}\text{D}4}^{+}$ Th cells, can be protective in immunosurveillance and are harmful in autoimmune illness. One of their distinguishing features is the production of interleukin (IL)-17 A, the classic effector cytokine of Th17, and IL-22, a member of the IL-10 family, under the effect of IL-6 and other cytokines [6]. IL-17 A and IL-22 are involved in the control of host defense against pathogens at barrier surfaces and tissue regeneration following injury [7, 8]. Autoimmune disorders, chronic inflammatory illnesses, and neoplasms have been linked to the pathophysiology of inappropriate Th17 activity and excessive production of IL-17 A or IL-22 [8, 9]. In the brain, Th17 cytokines can compromise the BBB, allowing peripheral immune cells and other factors into the CNS and promoting neuroinflammation and degeneration [10]. Th17 cytokines can create a propagative inflammatory cycle by activating granulocytes from the periphery and microglia in the brain to produce more inflammatory cytokines and chemokines [9]. Dysregulation of Th17 cells and their cytokines has been associated with several brain disorders like multiple sclerosis, experimental autoimmune encephalomyelitis, epilepsy, and ischemic brain injuries [11–13]. It has also been implicated in ASD [9]. Thus, patients with Th17-dominant autoimmune and neurological disorders may benefit from targeting the Th17 pathway or altering the biological activity of Th17 cytokines [14].

Numerous studies have looked into the potential link between IL-17 A and ASD as well as the severity of behavioral symptoms; IL-22 has not been included in many of them. In the current study, the serum levels of IL-17 A and IL-22 were measured in children diagnosed with ASD, and their correlation with the severity of the condition was assessed.

## Methodology

### Ethical considerations

The Research Ethics Committee of Ain Shams University Hospitals gave permission for this study (FAMSU MS 291/2022), and before its beginning, participants’ parents or caregivers provided written informed consent.

### Study settings and subjects

This pilot study was carried out over six months, in which we included 24 pediatric patients with ASD recruited from the Developmental and Behavioral Pediatric Clinic, Department of Pediatrics, Faculty of Medicine, Ain Shams University, during their follow-up visits. The diagnosis of autism followed the 5th edition of the Diagnostic and Statistical Manual of Mental Disorders (DSM-5) [15]. Patients with associated immunological, autoimmune, allergic, inflammatory, and other neuropsychiatric manifestations, and concurrent infections were excluded from the study. The control group comprised 24 apparently healthy children of similar ages and genders, not siblings of autistic patients.

### Study tools

The following was applied to each participant:

- **Detailed medical history from caregivers**, with particular emphasis on the developmental history, history of allergic or autoimmune manifestations, and the family history of allergic or autoimmune diseases.
- **Clinical examination**, including general, chest, cardiac, abdominal, and neurological assessments, to ensure the inclusion and exclusion criteria.
- **Neuropsychiatric assessment**, the degree of the disease severity was assessed using the Childhood Autism Rating Scale (CARS) [16], which rates the child on a scale from one to four in each of fifteen behavioral items. Scores up to 30 indicate mild autism, above 30 to 37 indicate moderate autism, and from 37 to 60 indicate severe autism.
- **Assessment of IQ level**, using full-scale IQ (FSIQ) [17], derived from the sum of all tasks in the Stanford-Binet 5. The test encompasses both the verbal and nonverbal domains of cognitive ability and offers an overall overview of the current state of general intellectual functioning.
- **Assessment of serum levels of IL-17 A and IL-22**, three mL of whole blood samples were withdrawn from all participants under complete aseptic conditions into a plain vacutainer tube containing gel and clot activator for serum separation by centrifugation at 3000 RPM for 10 min after whole blood clotting for 20 min. Separated sera were kept at -80°C until the time of analysis of IL-17 A and IL22 by human enzyme-linked immune sorbent assay (ELISA) kits (Bioassay Technology LLC, china, cat.No. E0142Hu and E0038Hu) The results were plotted on standard curves. The kit detection range of IL-17 A was from 2 pg/mL to 600 pg/mL, and the minimum detectable level of human IL-17 A was 1.06 pg/mL. Meanwhile, the kit detection range of IL-22 was from 5 pg/mL to 1500 pg/mL, and the minimum detectable level of human IL-22 was 2.48 pg/mL.

### Statistical analysis

The results were analyzed using Statistical Package for Social Sciences (SPSS) version 21 (IBM Corporation, NY, USA). Categorical variables were expressed as numbers and percentages. Numerical parametric variables were presented as a mean and standard deviation (± SD), while the numerical non-parametric variables were presented as the median and interquartile range (IQR). The Chi-square, Mann-Whitney, Kruskal-Wallis, and One Way ANOVA tests were used for data comparison. The Spearman correlation coefficient was used in correlations (r). The predictive performance of serum IL-17 A and IL-22 between ASD and control was determined using the receiver operating characteristic (ROC) curve. Logistic regression models were used to identify independent risk factors for ASD. Statistical significance is indicated by a *p*-value less than 0.05.

## Results

Forty-eight pediatric subjects participated in the current study, they were divided into 24 pediatric patients with ASD and 24 age- and gender-matched controls. The median (IQR) age of the ASD patient group was 5.25 (5–8) years and ranged from 3.5 to 14 years, while the median (IQR) age of the control group was 6 (5–9) years and ranged from 4 to 13 (*p*-value = 0.476). There was a male predominance in both ASD patient and control groups, with no significant difference between both groups (18 (75.0%) vs. 15 (62.5%); *p*-value = 0.350). The study groups had no significant differences regarding their family history of allergy and autoimmune diseases (*p*-values = 0.312 and 0.149, respectively).

In the ASD patient group, the mean (± SD) of the initial assessment CARS scores was 33.46 (± 4.83). Mild disorder was defined among six patients (25%), moderate was among 13 patients (54.2%), and severe was among five patients (20.8%). The mean (± SD) score of the IQ test of our ASD patients was 61.58 (± 9.30), ranging between 40 and 80. Therefore, cognitive impairment (IQ < 70) was defined among 79.2% of the patients, and five patients (20.8%) had below-average intelligence. Electroencephalogram (EEG) reported and confirmed convulsions in 3 of the included ASD patients (12.5%). They were well controlled on combined anti-epileptic therapies in the form of valproate, carbamazepine, and levetiracetam (Table 1).

**Table 1:** Clinical, demographic, and radiological data of the included autism spectrum disorder (ASD) patients (n = 24)

		ASD Patients
		n = 24
Type of Autism	Regressive	0 (0.0%)
	Primary	24 (100.0%)
Childhood Autism Rating Scale (CARS)	Mean ± SD	33.46 ± 4.83
	Range	25–44
Severity groups according to CARS scale	Mild up to 30	6 (25.0%)
	Moderate 30–37	13 (54.2%)
	Severe 37–60	5 (20.8%)
Score of intelligence quotient (IQ) test	Mean ± SD	61.58 ± 9.30
	Range	40–80
	Below average intelligence	5(20.8%)
	Cognitive impairment	19 (79.2%)
Electroencephalogram (EEG) abnormalities	Negative	21 (87.5%)
	Positive	3 (12.5%)
Presence of convulsions	No	21 (87.5%)
	Yes	3 (12.5%)
Frequency of convulsions	Irregular	2 (66.7%)
	Every 3 months	1 (33.3%)
Controlled convulsions	Yes	3 (100.0%)

**Fig. 1:**
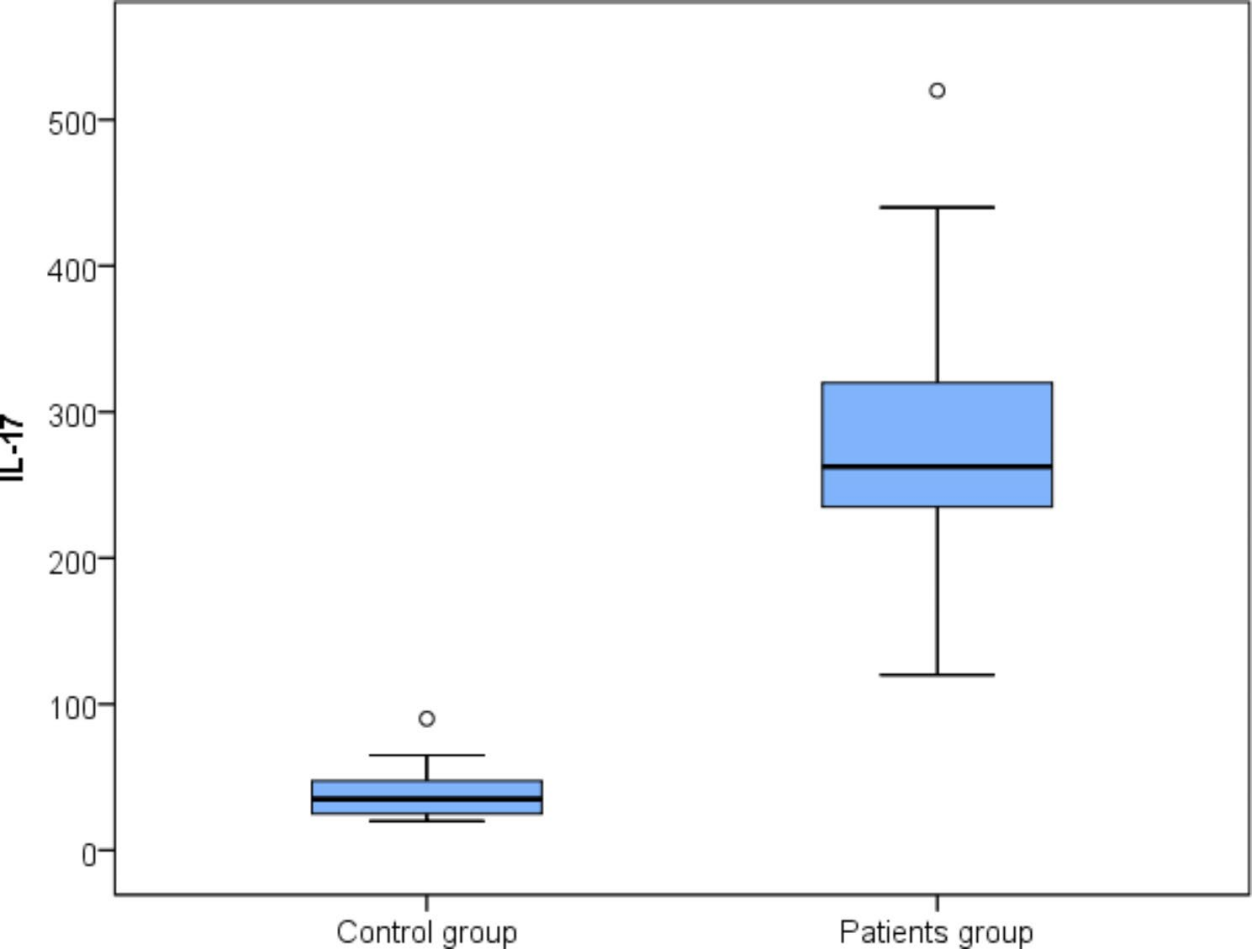
Box plot representing serum levels of interleukin (IL)-17 A in autism spectrum disorder (ASD) patients and controls (*p*-value < 0.001)

**Fig. 2:**
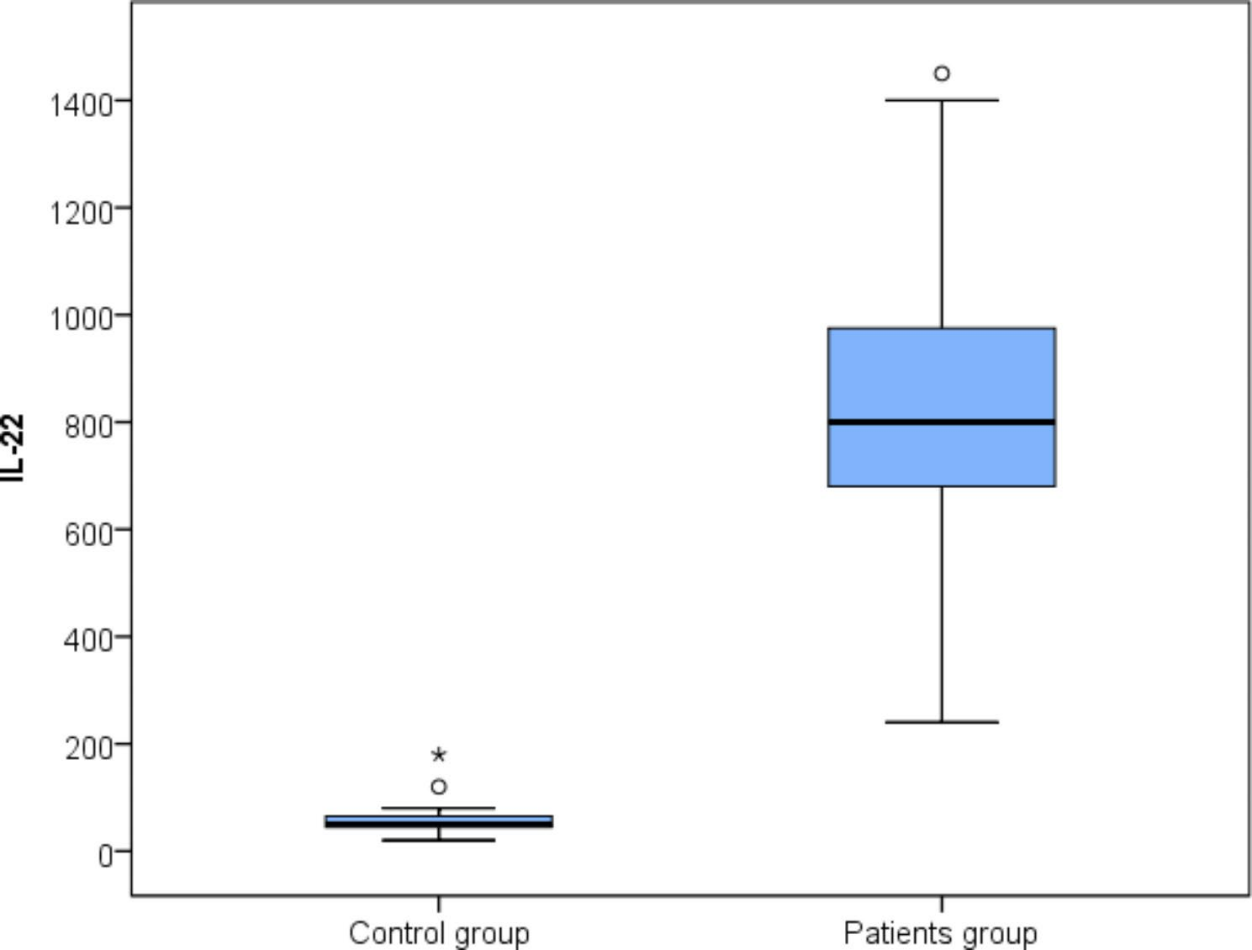
Box plot representing serum levels of interleukin (IL)-22 in autism spectrum disorder (ASD) patients and controls (*p*-value < 0.001)

The median serum levels of IL-17 A and IL-22 were significantly higher in ASD patients than in controls; the median (IQR) serum level of IL-17 A was 262.5 (235–320) pg/mL in ASD patients vs. 35 (25–47.5) pg/mL in controls; meanwhile the median (IQR) serum level of IL-22 in ASD patients was 800 (680–975) pg/mL vs. 50 (45–65) pg/mL in controls (*p*-values < 0.001) (Figs. 1 and 2).

Among the studied ASD patients, serum levels of IL-17 A and IL-22 showed no significant differences according to patients’ gender, presence of convulsions and EEG findings (*p*-values > 0.05) (Table 2).

**Table 2:** Comparison of serum levels of IL-17 A and IL-22 according to gender, presence of convulsions and electroencephalogram (EEG) abnormalities among the studied autism spectrum disorder (ASD) patients (n = 24)

		IL-17 pg/mL	Test value	*p*-value	IL-22 pg/mL	Test value	*p*-value
		Median (IQR)			Median (IQR)		
Gender	Female	255 (160–380)	-0.536•	0.592	1110 (800–1150)	-1.708•	0.088
	Male	265 (250–320)			700 (660–900)		
Presence of convulsions	No	260 (250–320)	-0.132•	0.895	850 (700–1000)	-1.535•	0.125
	Yes	320 (210–320)			700 (400–700)		
Electroencephalogram (EEG) abnormalities	Negative	260 (250–320)	-0.132•	0.895	850 (700–1000)	-1.535•	0.125
	Positive	320 (210–320)			700 (400–700)		

**•: Mann-Whitney test**

When ASD patients’ different clinico-demographic, radiological data and serum levels of IL-17 A and IL-22 were compared according to the severity categories according to CARS scores, we could not find any significant differences (Table 3).

**Table 3:** Comparison of the clinical, demographic, laboratory and radiological data of the included autism spectrum disorder (ASD) patients (n = 24) according to the severity by Childhood Autism Rating Scale (CARS)

		Childhood Autism Rating Scale (CARS) severity			Test value	*p*-value
		Mild	Moderate	Severe		
		n = 6	n = 13	n = 5		
Age (years)	Median (IQR)	5.25 (5–10)	5 (5–8)	7 (6–8)	1.062≠	0.588
	Range	413	3.59	3.514		
Gender (n., %)	Female	3 (50.0%)	1 (7.7%)	2 (40.0%)	4.677*	0.096
	Male	3 (50.0%)	12 (92.3%)	3 (60.0%)		
Presence of convulsions (n., %)	No	6 (100.0%)	11 (84.6%)	4 (80.0%)	1.213*	0.545
	Yes	0 (0.0%)	2 (15.4%)	1 (20.0%)		
Frequency of convulsions (n., %)	Irregular	0 (0.0%)	2 (100.0%)	0 (0.0%)	3.000*	0.083
	Every 3 months	0 (0.0%)	0 (0.0%)	1 (100.0%)		
Controlled convulsions	Yes	0 (0.0%)	2 (100.0%)	1 (100.0%)	–	–
Family history of allergy (n., %)	No	6 (100.0%)	12 (92.3%)	5 (100.0%)	0.883*	0.643
	Yes (Asthma)	0 (0.0%)	1 (7.7%)	0 (0.0%)		
Family history of autoimmune diseases (n., %)	No	6 (100.0%)	11 (84.6%)	5 (100.0%)	1.846*	0.397
	Yes	0 (0.0%)	2 (15.4%)	0 (0.0%)		
Score of intelligence quotient (IQ) test	Mean ± SD	65.83 ± 8.82	62.69 ± 8.47	53.60 ± 8.59	3.011•	0.071
	Range	55–80	40–70	41–65		
Electroencephalogram (EEG) abnormalities (n., %)	Negative	6 (100.0%)	11 (84.6%)	4 (80.0%)	1.213*	0.545
	Positive	0 (0.0%)	2 (15.4%)	1 (20.0%)		
Interleukin (IL)-17 (pg/mL)	Median (IQR)	262.5 (250–320)	260 (220–300)	360 (320–380)	2.349≠	0.309
	Range	120–520	170–440	160–440		
Interleukin (IL)-22 (pg/mL)	Median (IQR)	750 (660–850)	700 (600–900)	1120 (920–1150)	4.448≠	0.108
	Range	240–1450	400–1100	700–1400		

*: Chi-square test; ≠: Kruskal-Wallis test; •: One Way ANOVA test

**Fig. 3:**
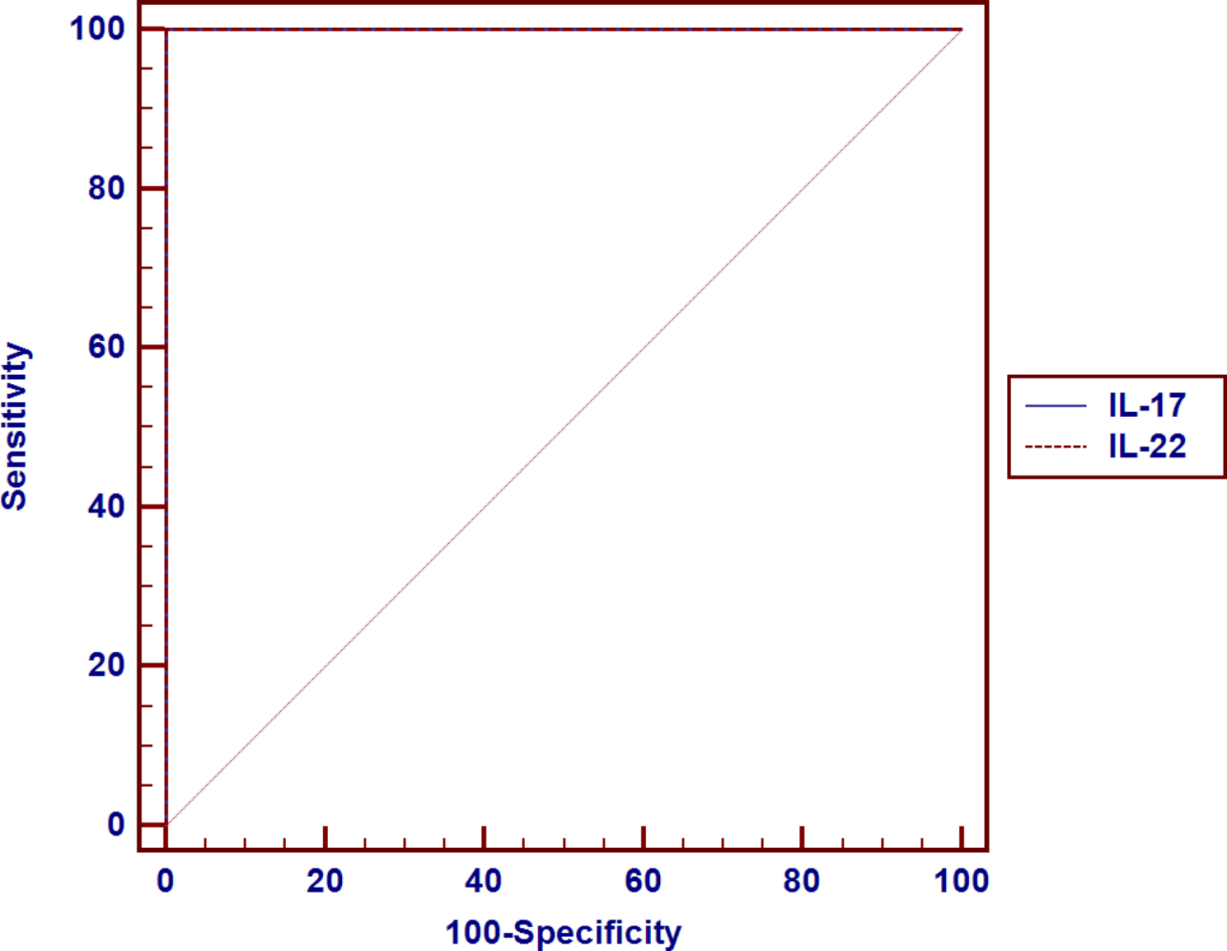
Receiver operating characteristic (ROC) curve of IL-17 and IL-22 to predict the autism spectrum disorder (ASD) between patients and controls

The best cutoff levels of serum IL-17 A and IL-22 to predict ASD according to the ROC curve analyses were > 90 pg/mL and > 180 pg/mL, respectively, with 100% diagnostic specificity, diagnostic sensitivity, positive predictive value, and negative predictive value (Fig. 3).

By logistic regression models, neither IL-17 A nor Il-22 were risk factors for ASD.

Although serum levels of IL-17 A and IL-22 were significantly positively correlated (r = 0.524, *p*-value = 0.009), only IL-22 had a significant positive correlation with ASD severity by CARS scores. IL-17 A and IL-22 showed no significant correlations with patients’ age and IQ scores (Table 4).

**Table 4:** Correlations of interleukin (IL)-17 A and IL-22 with age, Childhood Autism Rating Scale (CARS) scale and scores of intelligence quotient (IQ) test among the studied autism spectrum disorder (ASD) patients (n = 24)

	IL-17		IL-22	
	r	*p-value*	r	*p-value*
Interleukin (IL)-17 A (pg/mL)	–	–	0.524**	**0.009**
Interleukin (IL)-22 (pg/mL)	0.524**	**0.009**	–	–
Age (years)	0.219	0.305	0.067	0.757
Childhood Autism Rating Scale (CARS) scale	0.097	0.654	0.432**	**0.035**
Scores of intelligence quotient (IQ) test	0.116	0.589	-0.142	0.507

** Spearman correlation coefficient

## Discussion

ASD has been linked to several pathophysiological mechanisms. One of these mechanisms is immune system dysregulation, as seen by reports of elevated cytokine levels and inflammation [18]. Diagnosis of autism depends on behavioral features and developmental history because there are no available reliable biomarkers for the condition; behavioral therapy is the most widely used treatment for autism, and it works best when started early in childhood [19]. Due to the high associated public health costs, it is vital to develop diagnostic, treatment, and preventative measures for ASD [20].

According to Onore et al., 2009, cytokine analysis may identify aberrant immune responses as a biological characteristic marker of ASD [21]. Research has demonstrated that IL-17 A contributes, together with IL-22, to tissue regeneration following damage by inducing an acute immune response at mucosal and epithelial barriers, hence aiding in host defense against pathogens and the production of antimicrobial peptides. IL-17 A and IL-22 also have been reported to coordinate the interactions between the immunological, endocrine, and neurological systems. However, their excessive and uncontrolled production can induce several diseases [22, 23].

The current study revealed that the serum levels of IL-17 A and IL-22 were significantly higher in ASD patients than in controls. Only IL-22, but not IL-17 A, was significantly positively correlated with CARS scores but showed no significant difference according to ASD severity categories; the relatively small sample size may be the cause. The CARS scores of our included ASD patients ranged between 25 and 44, with a mean (± SD) value of 33.42 ± 4.61, approximating the recorded scores of previous studies by Perry et al.,2005, and Zahra et al.,2022, (36.1 ± 4.6 and 36 ± 5.2, respectively) [24, 25].

### Serum IL-17 A and ASD

IL-17 A may play a role in several autoimmune neuroinflammatory illnesses as it promotes other proinflammatory cytokines that generate the Th1 response [26]. In addition, IL-17 A may induce brain auto-antibodies and neutrophil-recruiting chemokines [27]. Furthermore, uncontrolled expression of IL-17 A may disrupt the BBB and neural connectivity by acting on astrocyte and microglia receptors. Thus, it has been linked to several CNS disorders [28].

Moaaz et al., 2019, showed that the Th17/T-regulatory (Treg) cell ratio in children with ASD was biased toward a Th17 response in comparison to their control group and that Th17 cells and Th17/Treg cell ratio were significantly positively correlated with ASD severity [29]. Furthermore, Arteaga-Henríquez et al., 2023, demonstrated that different immunoregulatory agents could improve symptoms of ASD by restoring the Th17/TTreg cell ratio imbalance and decreasing the levels of proinflammatory cytokines like IL-17 A, both in the blood and in the brain of individuals with ASD [30].

Several other studies reported that the levels of IL-17 A were significantly elevated in patients with autism compared to controls [27, 31, 32]. A study in 2012 [27] found serum IL-17 A significant elevation in about half of their included patients with autism, which correlated with the severity of the disease. In addition, Akintunde et al., 2015, [32] measured IL-17 levels in activated cell supernatants, rather than serum levels, after ex vivo phytohemagglutinin mononuclear cell stimulation. They found that cells from patients with ASD had increased production of IL-17 compared to controls, but the levels of IL-17 did not correlate with disease severity scores. Similarly, Suzuki et al., 2011, [31] could not find any association between the elevated levels of IL-17 A in ASD patients and disease severity. They hypothesized this elevation might be due to an aberrant steady-state immune response in ASD patients.

### Association with autoimmune/inflammatory disorders

In the current study, the exclusion criteria included ASD patients with associated autoimmune, allergic, and inflammatory manifestations, as well as concurrent infections because studies indicate a higher incidence of immune-related disorders, such as allergies, viral infections, primary immunodeficiency, and autoimmune disorders, in individuals with ASD and their families [33, 34]. A study on murine by Gumusoglu et al., 2020, found that maternal IL-17 A levels during gestation correlated with the neurodevelopmental and behavioral phenotypes relevant to ASD [20]. Another study by Fujitani et al., 2022, [35] discussed the effect of human maternal immune activation by infection or molecular mimicry as a risk factor for ASD. They reported that the provoked maternal IL-17 A resulted in brain abnormalities, abnormal behavior, and ASD in offspring. They also hypothesized that blocking of IL-17 A production in mothers could alleviate ASD symptoms in offspring. In the current study, the family history of autoimmune disorders and allergic diseases was demonstrated in 8.3% and 4.2% of our studied ASD patients, respectively; however, it was statistically insignificant in comparison to controls. It was reported previously by Croen et al., 2019, [36] that the maternal history of allergy/autoimmune diseases increased the risk of ASD in their offspring by 40%. Furthermore, Khakzad et al., 2012, [37] hypothesized that the aberrant immune responses in the form of either allergic reactions or autoimmune disorders in the mothers during pregnancy would impact fetal brain development. They reported a two-fold increased risk of ASD among children whose mothers suffered from allergies in the second trimester of pregnancy [37, 38].

On the other hand, several other studies did not reveal any association between serum IL-17 A levels and autism [21, 39–41]. The results discrepancies could be attributable to different cohort sizes, participants’ ages, and cytokine detection methods. Liu et al., 2014, [42] reported that IL-17 A is a negative regulator of neurogenesis, and knocking out IL-17 A enhances synaptic function.

### Serum IL-22 and ASD

The function of IL-22 in CNS disorders is controversial. Mattapallil et al. 2019, [43] reported that IL-22 has a neuroprotective and regenerative role in the CNS. On the other hand, according to Chen et al. 2022 [44], IL-22 can disrupt the BBB and permit lymphocyte entry into the CNS, promoting neuroinflammation. Although research has linked IL-22 to the development and progression of several neurological and autoimmune disorders like inflammatory myopathies, myasthenia gravis, systemic lupus erythematosus, rheumatoid arthritis, Sjogren’s syndrome, psoriasis, Crohn’s disease, multiple sclerosis, Alzheimer’s disease, encephalitis, and Guillain-Barré syndrome [45], few reports discussed the involvement of IL-22 in ASD and no prior studies have investigated the correlation between IL-22 and CARS scores or ASD severity categories. Robinson-Agramonte et al. 2022, [46] reported that IL-17 A and IL-22 from the brain–peripheral interactions can affect brain development, neuronal function, and behavior in neurodevelopmental disorders. They noted that autistic children showed dysregulation of T-cell activities with a skewed CD4/CD8 ratio, which could result in increased cytokine production, including IL-17 A and IL-22, and decreased executive function. They stated that the Th1 endophenotype correlated with more severe ASD behavioral symptoms. In addition, Ahmed et al. 2017, [47] found that IL-22 expression was higher in autistic children and concluded that IL-22 induction might contribute to ASD and link immunological and neural dysfunctions in autism. Another study by Aldossari et al., 2023, investigated the expression levels of several inflammatory mediators and transcription factors by CD40^+^ cells in children with ASD using a flow cytometric analysis because the CD40 signaling pathway in antigen-presenting cells can promote several biological functions, including inflammatory cell polarization, cytokine release, and immunoglobulin isotype switch. They found that IL-17 A- and IL-22 expressing CD40^+^ cells were present in significantly higher numbers among ASD children compared to controls, but they were not correlated with ASD severity [48].

### Association with epilepsy and IQ scores

In the current study, we had three autistic children (12.5%) with medically controlled epilepsy. EEG findings were in the form of left centroparietal spikes and right temporal spikes. According to Ewen et al., 2019, [49] the frequency of epilepsy among children with ASD was 9.1 to 9.8%. In addition, Mostafa et al., 2015, [50] reported epilepsy among 7% of autistic children. They also noted that EEG activities were frequently elicited among autistic children, with or without convulsions (40% and 44%, respectively).

Moreover, the IQ scores of our studied ASD patients ranged from 40 to 80, with only five patients (20.8%) recorded below-average intelligence and cognitive impairment (IQ < 70) was detected among 19 patients (79.2%). Similarly, Charman et al., 2011, [51] reported that 55% of their autistic patients had intellectual disabilities with IQ scores < 70 and only 16% had moderate to severe intellectual disabilities with IQ scores < 50. The imbalanced neurotransmitters could explain the functional and cognitive abnormalities associating ASD.

The current study found no association between levels of IL-17 A or IL-22 and convulsion, EEG abnormalities or IQ test scores. This is the opposite of what is expected because excessive production of pro-inflammatory cytokines and systemic inflammation can enhance the BBB’s permeability and the functional activity of brain-resident cells, both of which are responsible for inducing convulsions [52, 53]. Our findings could be due to the co-associated different genetic factors, metabolic disorders, vitamin and mineral deficiency or other immune cell dysfunctions that could influence the electrochemical activity of the brain cells [54].

### Study limitations

Previous research indicated that the intestinal microbiome could play a crucial role in regulating immune responses and CNS functions. Changes in human intestinal microbial flora, dysbiosis, and gastrointestinal inflammation could also lead to autism development and affect its phenotypes [55, 56]. The current study did not look into this link as well as into the history of breastfeeding, which is considered a major limitation of it. Stratifying ASD patients according to these factors could potentially unmask a niche of ASD phenotypes more closely linked to such cytokines, thereby gaining more clarity on the relationship between inflammation, immunity, and autism. The relatively small sample size in the current study is also a significant limitation. Further extensive studies will be needed that include more advanced techniques for multiplex cytokine detection with long follow-up periods to evaluate better serum IL-17 A and IL-22 levels and functions in pediatric patients with autism and to confirm whether IL-22 correlates to ASD severity.

## Conclusion

Despite much research has investigated the association between IL-17 A levels and ASD, few looked into the link with IL-22. The current study suggests that elevated levels of IL-17 A and IL-22 are associated with ASD, and specifically, IL-22 is related to the disease severity by CARS scores, recommending that IL-22 could be a potential future more effective therapeutic target for ASD. Further research, with an increased number of cases, is needed to understand better these cytokines’ role in autism and their potential implications for diagnosis and treatment. As well as to identify the particular driver of inflammation associating ASD.

## Data Availability

On reasonable request, the corresponding author will provide the datasets used and/or analyzed during the current work.
